# How Integrated Management Strategies Promote Protein Quality of Cotton Embryos: High Levels of Soil Available N, N Assimilation and Protein Accumulation Rate

**DOI:** 10.3389/fpls.2016.01118

**Published:** 2016-08-02

**Authors:** HongKun Yang, YaLi Meng, BingLin Chen, XingYue Zhang, YouHua Wang, WenQing Zhao, ZhiGuo Zhou

**Affiliations:** Key Laboratory of Crop Physiology and Ecology, Ministry of Agriculture, Nanjing Agricultural University and Jiangsu Collaborative Innovation Center for Modern Crop ProductionNanjing, China

**Keywords:** cotton embryonic protein, integrated management strategies, soil available N, N assimilation, proteinogenic amino acids

## Abstract

Cottonseed is widely used as a source of ruminant feed and for industrial purposes. Therefore, there is a tremendous need to improve the nutritional value of cotton embryos. In this study, a conventional management (CM) and two integrated cotton management strategies (IMS_1_, IMS_2_) were performed at two soil fertility levels to study the relationships among soil N, N assimilation, embryonic protein accumulation and protein quality. The levels of proteins, essential amino acids, and semi-essential amino acids, especially those of glutamate, lysine, and methionine, were higher in IMS_1_ and IMS_2_ embryos than in CM embryos. These changes were significantly positively correlated with the soil-available N content, glutamine synthetase activity and peak value of protein accumulation rate and were negatively correlated with the free amino acid level. These results illustrated that integrated management strategies, especially the rates and timing of N application, raise the level of soil available N, which is beneficial for N assimilation in developing cotton embryos. The protein content was limited by the rate of protein accumulation rather than by the free amino acid content. The combination of target yield fertilization, a growth-driven N application schedule, a high plant density and the seedling raising with bio-organic fertilizer can substantially improve protein quality in cotton embryos, especially at a soil with low soil organic matter and total nitrogen.

## Introduction

Cotton embryos accumulate 40%–55% of their dry weight (DW) as storage protein, which has a substantial effect on their nutritional value (Alford et al., 1996; Mujahid et al., 2000). Therefore, evaluating the mechanisms controlling storage protein accumulation and protein quality in these embryos is crucial step for improving seed quality. Recently, considerable progress has been made in elucidating the relationships among genotypes, environmental conditions and management practices, suggesting that protein content is a variety-specific quantitative characteristic (Yu et al., 2012) and that it is substantially influenced by environmental conditions (Li et al., 2009; Rotundo and Westgate, 2009) and crop management strategies (Sawan et al., 1993, 2001, 2006, 2009). Numerous studies show the many steps involved in protein accumulation in cottonseed embryos, involving seed biochemistry (Hodges, 2002; Hernandez-Sebastia et al., 2005; Zhang et al., 2014), N transport with the plant and from the soil (De Ruiter et al., 1986; Kullmann and Geisler, 1986; Crawford and Glass, 1998), soil N levels and the environment (Macduff and Hopper, 1986). Integration of the results of these studies into a complete management system targeting the final result of protein quality in cotton embryos is still needed.

Integrated management strategies are combinations of agronomic practices applied to achieve high yields, optimal quality, and long-term sustainability based on analyses of factors limiting the productivity and quality of crops under a given set of environmental conditions. The planting of high-yielding cultivars with optimal nutritional management (Sapkota et al., 2014) and water management strategies (Falkenberg et al., 2007), adequate plant populations (Dong et al., 2012), and strong seedlings (Mcgraw and Shaver, 2006) are major requirements for high yields and optimal quality. Integrated nutrient management improves the soil productivity, crop yield and quality of groundnuts (Prasad et al., 2002) and tomatoes (Javaria and Khan, 2011). Whether IMS can also improve protein quality in cotton embryos requires further elucidation.

A considerable amount of data is available regarding the “source-sink” relationships in the study of embryonic protein synthesis (Martre et al., 2003; Seong et al., 2004; Zhang et al., 2012), whereas the relationships between soil N and embryonic proteins are unclear. AV-N (i.e., the sum of 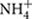 -N and 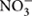 -N), as an indicator of soil N and fertility, can be directly utilized by crop roots (Crawford and Glass, 1998) and ultimately incorporated into storage protein (Kullmann and Geisler, 1986). Soil N increased with increasing fertilization rate (Sainju et al., 2006). Integrated nutritional management (Javaria and Khan, 2011; Singh et al., 2014) and soil temperature (Macduff and Hopper, 1986) can influence seasonal and regional variations in the soil available N. Moreover, soil N also regulates N uptake and plant growth (Gastal and Lemaire, 2002). Therefore, it is necessary to explore the relationship between soil N and protein content.

Some of the crucial steps in initial N assimilation are the incorporation of N into carbon skeletons of storage proteins and its use for the biosynthesis of glutamine and then of Glu (Hodges, 2002). Glutamine synthetase (GS) and glutamate synthase (GOGAT) play a key role in N assimilation for its higher affinity to 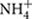 -N than glutamate dehydrogenase (GDH) (Stewart and Rhodes, 1978; Hodges, 2002; Hodges et al., 2003; Muro-Pastor and Florencio, 2003; Muro-Pastor et al., 2005; Potel et al., 2009). N assimilation uses ferredoxin (Fd-GOGAT, EC 1.4.7.1) as an electron carrier in photorespiratory tissues (i.e., leaf samples) and NADH (NADH-GOGAT; EC 1.4.1.14) as an electron carrier in non-photorespiratory tissues (i.e., roots and seeds) (Canovas et al., 1998; Tamura et al., 2010; Konishi et al., 2014). The 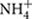 -N and 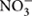 -N concentrations in the soil tightly regulate the GS and GOGAT activities (Zhao and Shi, 2006). Although the functional roles of GS (EC 6.3.1.2) and GOGAT (EC 1.4.1.14) in plant roots and leaves have been well described (Ishiyama et al., 1998; Muro-Pastor and Florencio, 2003; Potel et al., 2009), the role of nitrogen assimilation in non-photosynthetic organs is unclear, especially in protein-enriched cotton embryos.

Cotton embryos are characterized by high protein content and are rich in Lys and Glu, which serve as nutritional limiting factors for ruminants (Bertrand et al., 1998; Zhe et al., 2014). The globulin null soybean mutant accumulates high levels of free amino acids (Takahashi et al., 2003), suggesting a role of free amino acids in storage protein accumulation. Varietal and locational factors may influence the contents of free amino acids and storage protein in cottonseeds (Ikurior and Fetuga, 1987). A fundamental understanding of the conversion of free amino acids into storage proteins and the characterization of protein accumulation would clarify the factors limiting the protein accumulation rate.

The objective of the present study was to improve protein quality in cotton embryos by adopting IMS and to analyze how these strategies improve protein quality in terms of soil N, N assimilation, the protein accumulation rate, proteinogenic amino acids, and the protein content. The initial hypothesis was that the combination of optimal N management practices, an adequate plant density and strong seedlings could be beneficial to protein quality in cotton embryos.

## Materials and Methods

### Growth Conditions and Integrated Management Strategies

Field experiments were carried out at the Dafeng Experimental Station, Jiangsu, China (120°45′ E, 33°19′ N), using a widely grown cotton cultivar, Siza-3 (*Gossypium hirsutum* L.), in 2012 and 2013. The soil at the experiment site was a typical sandy loam, and there were significant differences in the contents of organic matter and total nitrogen (**Table 1**), with low-fertility soil containing an average of 14.31 ± 0.62 g kg^-1^ soil organic matter and 0.79 ± 0.04 g kg^-1^ total nitrogen and high-fertility soil containing an average of 17.76 ± 0.69 g kg^-1^ soil organic matter and 0.86 ± 0.03 g kg^-1^ total nitrogen at a 0–20 cm depth of the soil profile.

**Table 1:** Soil nutrient status at the experimental site in 2012 and 2013.

Year	Soil fertility	Soil depth (cm)	Bulk density (g m^-3^)	pH	SOM (g kg^-1^)	WSOC (mg kg^-1^)	TN (g kg^-1^)	Av–N (mg kg^-1^)	Av–P (mg kg^-1^)	Av–K (mg kg^-1^)
2012	LF	0–20	1.20	8.47	14.80	46.70	0.79	23.90	19.00	364.50
		20–40	1.51	8.55	12.60	42.80	0.67	18.00	14.20	273.40
	HF	0–20	1.16	8.26	18.30	51.20	0.86	28.60	23.50	384.00
		20–40	1.47	8.34	15.70	46.50	0.73	21.40	17.60	288.10
2013	LF	0–20	1.17	8.49	13.60	45.90	0.73	22.20	17.30	394.50
		20–40	1.46	8.51	11.90	41.80	0.69	19.70	15.60	300.70
	HF	0–20	1.11	8.21	17.20	50.70	0.82	27.30	22.20	414.20
		20–40	1.39	8.47	14.00	46.40	0.71	23.60	19.30	316.80


*LF: low soil fertility level; HF: high soil fertility level; SOM: soil organic matter; WSOC: water soluble organic carbon; TN: soil total nitrogen; Av–N: soil available nitrogen; Av–P: soil available phosphor; Av–K: soil available potassium.*

Cotton plants were planted using a CM system and two integrated management strategies (IMS_1_ and IMS_2_). A randomized complete block design with three replicates was used in a plot measuring 22 m long and 10 m wide. CM was a widely used practice in the Yangtze River cotton-producing region (i.e., 300 kg N ha^-1^ + 18,000 plants ha^-1^ + seedling transplantation, with 40% of the 300 kg N ha^-1^ applied as a basal fertilizer and 60% applied at the initial flowering stage). The two integrated management strategies (IMS_1_ and IMS_2_) included different combinations of N rates, N application schedules, plant densities, and seedling-raising methods. IMS_1_ included an economic N rate, a high plant density and substrate seedling-raising method (i.e., 375 kg N ha^-1^ + 30,000 plants ha^-1^ + substrate seedling raising). Compared to the CM, in IMS_2_, the N rate was increased to 525 kg N ha^-1^, the plant density was increased to 30,000 plants ha^-1^, and seedling transplantation was adopted (i.e., target yield fertilization + higher plant density + seedling transplantation).

Target yield fertilization and the economic N rate were calculated according to a previously described algorithm (Katayama et al., 1999; Baker and Young, 2004). The N application schedules of IMS_1_ and IMS_2_ were timed to coincide with growth-driven N demand of cotton plants, i.e., 20% applied as a basal fertilizer, 25% at the initial flowering stage, 40% at the full-bloom stage and 15% at the end of the flowering stage. N, P, and K were applied as urea (46% N), ordinary superphosphate (12% P_2_O_5_ and 12% sulfur) and potassium sulfate (50% K_2_O and 18% sulfur) at a ratio of 1.0:0.6:1.1. For substrate seedling raising, seeds were planted with bio-organic fertilizer (20 million g^-1^ efficacious living-cell and 43.6% organic matter, purchased from Jiangsu Tianniang Ltd., China) at the rate of 10 g per seedling, and seedlings were transplanted in the field with a bio-organic fertilizer on May 15th (3–4 true leaves). For seedling transplantation, cottonseeds were planted in a nursery bed, and then seedlings with 3–4 true leaves were transplanted in the field without bio-organic fertilizer on May 15th. Integrated pest management and furrow irrigation were applied to avoid any biotic and abiotic stresses during the cotton growth period.

### Sampling

White flowers from first fruiting nodes at the 7th–8th sympodial branches were tagged with the flowering date to ensure that boll samples in each treatment were collected at equivalent metabolic and developmental boll ages. Tagged boll samples were collected once every 7 days from 17 days after anthesis (DAA) until boll opening from 9:00 to 10:00 a.m. local time. Collected bolls were quickly separated into fibers, carpels, seed coats and embryos at 4°C. About half of the embryo samples were placed in liquid nitrogen and stored at -80°C for assays of GS and NADH-dependent GOGAT. The other half of embryo samples were dried to a constant weight, and ground to pass through a 1 mm sieve, and then used to determine the free amino acid and protein contents, as well as the compositions of proteinogenic amino acids.

### Soil Available Nitrogen

Fresh and uniformly mixed soil samples collected from 0–20 cm to 20–40 cm depths in each plot were assayed immediately. Soil samples (5 g) were extracted with 50 ml of 0.01 M CaCl_2_ for 1 h using a shaking table. A continuous flow analyser (Bran and Luebbe TRAACS Model 2000 Analyzer) was used for determinations of the soil nitrate-nitrogen (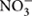 -N) and ammonium nitrogen (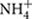-N) levels (Dahnke and Johnson, 1990). The soil-available nitrogen level was calculated as the sum of the nitrate-nitrogen (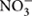 -N) and ammonium nitrogen (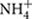 -N) levels.

### GS and NADH-Dependent GOGAT Assays

The extraction and measurement of GS (EC 6.3.1.2) and NADH-GOGAT (EC 1.4.1.14) were performed according to the method of Lea et al. (1990). Frozen embryo tissues (0.3 g) were homogenized in 5 ml buffer containing 50 mM KH_2_PO_4_ (pH 7.2), 2 mM EDTA, 2 mM DTT, 5% (v/v) glycerol, and 1% (w/v) insoluble PVPP. After centrifugation at 12,000 × *g* for 20 min at 4°C, the supernatant homogenates were stored at 4°C for assays of GS, NADH-dependent GOGAT and soluble protein, which were conducted immediately.

#### GS Assay

The standard assay mixture (1.6 ml) contained 0.15 mM imidazole buffer (pH 7.0), 0.010 mM MgSO_4_, 0.12 mM Glu-Na, and 0.012 mM ATP-Na. After adding 1.2 ml crude enzyme and heating for 5 min at 25°C, the reaction was initiated by 0.2 ml of 1 M NH_2_OH for 15 min at 25°C. The amount of γ-glutamyl hydroxamate (GH) generated was determined by adding 0.8 ml ferric chloride reagent (0.37 mM FeCl_2_, 0.67 mM HCl and 0.2 mM trichloroacetic acid) and spectrophotometrically measuring absorbance at 540 nm. The activity of GS is expressed as micromoles of γ-glutamyl hydroxamate formed per milligram of soluble protein per hour.

#### NADH-GOGAT Assay

NADH-dependent GOGAT was assayed at 340 nm by coupling the reaction to NADH oxidation mediated by reductive amination of α-ketoglutarate at saturating substrate concentrations (Muhitch, 2006). The reaction mixture contained 0.1 M Tris-HCl (pH 8.5), 0.2 M α-ketoglutarate, 1.0 mM CaCl_2_, and 0.2 mM NADPH. After heating to 25°C, the reaction was initiated by adding monosodium Glu to a final volume 2.1 ml. After incubating the mixture in a water bath at 30°C for 30 min, the rate of decline in the NADH concentration was determined using a spectrophotometer at 340 nm. The activity of GOGAT is expressed as micromoles of NADH per milligram of soluble protein per hour.

#### Soluble Protein Assay

Soluble protein was quantified using the Bradford protocol (Bradford, 1976). The standard mixture contained 0.01% (w/v) Coomassie brilliant blue (G-250), 4.7% (w/v) ethanol and 8.5% (w/v) phosphoric acid. After adding 50 μL of extract to the standard mixture in a total volume of 5 ml, the contents were mixed thoroughly, and absorbance was spectrophotometrically measured at 595 nm against a blank reagent at the indicated time points, ranging from 2 to 30 min. Soluble protein was quantified using a standard soluble protein (Bovine Serum Albumin).

### Free Amino Acid Content

Free amino acids were extracted from dried and powdered cotton embryo tissues (0.1 g) with 5 ml of 80% ethanol in a water bath at 80°C for 30 min (Hendrix, 1993). After three rounds of extraction, the samples were diluted to a final volume of 25 ml. Assays were performed in a 96-well polystyrene plate with a Benchmark microplate reader (Bio-Rad). The free amino acid content is expressed as mg g^-1^ DW.

### Protein Content and Proteinogenic Amino Acid Profiles

Protein content was calculated as the product of the 100-embryo weight (g), N concentration (%) and 6.25. Proteinogenic amino acids were isolated from dried and powdered cotton embryos (30 mg) via acidolysis with 10 ml of 6 M HCl for 24 h at 110°C. The solution was diluted to a final volume of 50 ml with 0.02 M HCl. Profiles of proteinogenic amino acids were determined using an automatic amino acid analyzer (L8900, Hitachi, Tokyo, Japan) (Hao et al., 2014).

A sigmoid growth curve was used to assess the accumulation of storage proteins in developing cotton embryos, where embryo protein is the protein content (g 100 embryos^-1^) at developmental time (d), *P*_max_ is the protein content at maturity, a and b are constants, DAA_1_ is the start time of rapid embryo protein accumulation, and DAA_2_ is the termination time of rapid embryo protein accumulation. The duration is the difference (in days) between the two dates.

$${Embryo\,\;protein}_{\left(t\right)}=\frac{P_{max}}{1+{ae}^{b\times DAA}}$$

$${Rate}_{\left(t\right)}=\frac{P_{max}ab\times e^{b\times DAA}}{{\left(1+{ae}^{b\times DAA}\right)}^{2}}$$

$$Duration={DAA}_{2}-{DAA}_{1}=\frac{1}{b}In\frac{2+\sqrt{3}}{a}-\frac{1}{b}In\frac{2-\sqrt{3}}{a}$$

### Statistical Analysis

Three-way analysis of variance was performed on at least three replicates to examine the effects of years, fertility levels, IMS and their interactions on the composition of proteinogenic amino acids. The measurements are expressed as the mean ± SE. Fisher’s least significant difference (LSD) test was used for statistical analyses. Statistical significance is indicated by *P* < 0.05 or by *P* < 0.01. SAS software was used for principal component analysis (PCA), and graphs were plotted using Origin software, version 9.0.

## Results

### Protein Quality in Cotton Embryos

When seeds were mature, the cotton embryos accumulated 42.7–46.9% of their DW as storage protein. The protein contents ranged (g 100 embryos^-1^) from 2.18–2.68 g in 2012 to 2.63–3.30 g in 2013 (**Table 2**). The protein contents in the IMS_1_ and IMS_2_ embryos were 14.5 and 24.3% higher at low soil fertility levels and 3.3 and 12.7% higher at high soil fertility levels, respectively, compared to those in the CM embryos (*P* < 0.05).

**Table 2:** Mean protein content (g 100 embryos^-1^) of recommended integrated crop management (ICM) as compared with conventional management practice (CM) for cottonseed embryos in 2012 and 2013.

Year	Soil fertility	Integrated management strategies	N rates (kg ha^-1^)	N application schedule	Plant density (plant ha^-1^)	Seedling raising method	Protein content	Increase of IMS over CM (%)	*P*
2012	LF	CM	300	120-180-0-0	18,000	ST	2.01		
		IMS_1_	375	75-94-150-56	30,000	SR	2.38	18.38	<0.05
		IMS_2_	525	105-131-210-79	30,000	ST	2.65	32.16	<0.05
	HF	CM	300	120-180-0-0	18,000	ST	2.35		
		IMS_1_	375	75-94-150-56	30,000	SR	2.43	3.35	ns
		IMS_2_	525	105-131-210-79	30,000	ST	2.68	14.18	<0.05
2013	LF	CM	300	120-180-0-0	18,000	ST	2.63		
		IMS_1_	375	75-94-150-56	30,000	SR	2.91	10.56	<0.05
		IMS_2_	525	105-131-210-79	30,000	ST	3.06	16.47	<0.05
	HF	CM	300	120-180-0-0	18,000	ST	2.97		
		IMS_1_	375	75-94-150-56	30,000	SR	3.07	3.36	ns
		IMS_2_	525	105-131-210-79	30,000	ST	3.30	11.33	<0.01


*LF and HF represent the low soil fertility level and high soil fertility level, respectively. CM and IMS represent the conventional management practices and integrated management strategies, respectively. The N application schedules of IMS_1_ and IMS_2_ were timed to coincide with growth-driven N demand of cotton plants (see Materials and Methods). ST: Seeds were planted in a nursery bed, and then seedlings were transplanted into the field on May 15th. SR: Seeds were planted with bio-organic fertilizer, and seedlings were transplanted into the field with substrate on May 15th. Probability (P) of significant treatment difference according to a paired t-test; NS, not significantly different at *P* = 0.05.*

The protein content increased significantly with increases in the N rate and plant density (**Table 2**). The application of 20% N as basal fertilizer, 25% N at the initial flowering stage, 40% N at the full-bloom stage and 15% N at the end of the flowering stage showed advantages over the N application schedule of CM. The seedling-raising method also had beneficial effects on the protein content.

The acidolysis of proteinogenic amino acids from storage proteins indicated that the contents of total amino acids and each proteinogenic amino acid in the IMS_1_ and IMS_2_ embryos were increased compared to those in CM (**Table 3**). The contents of total proteinogenic, essential, semi-essential and non-essential amino acids in IMS_2_ increased by averages of 7.61, 8.04, 4.64, and 8.32%, respectively, whereas they did not significantly differ between IMS_1_ and CM. Although, the soil fertility level did not affect the composition of proteinogenic amino acids, the interaction effect between fertility level and IMS was significant (*p* < 0.05, **Table 4**), demonstrating IMS did not only affect proteinogenic amino acids profiles, but they also have a beneficial effect on soil fertility level.

**Table 3:** Proteinogenic amino acid contents of cotton embryos under conventional management practices and two integrated management strategies at two fertility levels.

Amino acids		Low fertility level (%)			High fertility level (%)		
		
		CM	IMS_1_	IMS_2_	CM	IMS_1_	IMS_2_
(a)							
NE	Asp	4.18 ± 0.18 bc	4.35 ± 0.16 ab	4.55 ± 0.03 a	4.10 ± 0.02 b	4.22 ± 0.08 b	4.42 ± 0.02 a
	Ser	2.02 ± 0.07 bc	2.10 ± 0.06 ab	2.19 ± 0.01 a	1.99 ± 0.00 c	2.05 ± 0.04 b	2.12 ± 0.01 a
	Glu	10.01 ± 0.42 bc	10.43 ± 0.36 ab	10.92 ± 0.03 a	9.84 ± 0.03 b	10.17 ± 0.22 b	10.65 ± 0.04 a
	Gly	1.88 ± 0.08 bc	1.94 ± 0.07 ab	2.02 ± 0.01 a	1.85 ± 0.01 b	1.91 ± 0.04 b	1.98 ± 0.01 a
	Ala	1.75 ± 0.06 bc	1.81 ± 0.06 ab	1.90 ± 0.01 a	1.72 ± 0.01 b	1.77 ± 0.04 b	1.85 ± 0.01 a
	Cys	0.71 ± 0.02 bc	0.74 ± 0.02 ab	0.77 ± 0.01 a	0.71 ± 0.01 b	0.72 ± 0.02 b	0.75 ± 0.01 a
	Tyr	1.32 ± 0.06 bc	1.37 ± 0.04 ab	1.43 ± 0.01 a	1.28 ± 0.02 c	1.37 ± 0.02 b	1.41 ± 0.01 a
	Pro	1.59 ± 0.05 bc	1.66 ± 0.06 ab	1.72 ± 0.01 a	1.55 ± 0.01 b	1.61 ± 0.04 ab	1.68 ± 0.02 a
Total %		23.46 ± 0.94 bc	24.40 ± 0.84 ab	25.50 ± 0.09 a	23.03 ± 0.03 b	23.83 ± 0.49 b	24.86 ± 0.12 a
(b)							
E	Thr	1.46 ± 0.06 bc	1.52 ± 0.05 ab	1.58 ± 0.01 a	1.43 ± 0.01 c	1.48 ± 0.03 b	1.54 ± 0.01 a
	Val	1.92 ± 0.08 bc	1.99 ± 0.08 ab	2.08 ± 0.01 a	1.88 ± 0.02 b	1.96 ± 0.04 b	2.05 ± 0.02 a
	Met	0.63 ± 0.01 bc	0.68 ± 0.03 ab	0.70 ± 0.01 a	0.63 ± 0.00 b	0.65 ± 0.02 ab	0.66 ± 0.00 a
	Ile	1.38 ± 0.05 b	1.42 ± 0.05 ab	1.49 ± 0.01 a	1.36 ± 0.01 b	1.40 ± 0.03 b	1.46 ± 0.01 a
	Leu	2.69 ± 0.09 bc	2.79 ± 0.10 ab	2.90 ± 0.01 a	2.64 ± 0.01 c	2.73 ± 0.05 b	2.84 ± 0.02 a
	Phe	2.61 ± 0.09 bc	2.71 ± 0.12 ab	2.84 ± 0.01 a	2.54 ± 0.01 c	2.65 ± 0.05 b	2.77 ± 0.01 a
	Lys	1.99 ± 0.07 ab	2.06 ± 0.08 ab	2.13 ± 0.02 a	1.95 ± 0.01 b	2.02 ± 0.04 b	2.10 ± 0.02 a
Total %		12.69 ± 0.46 bc	13.16 ± 0.51 ab	13.72 ± 0.04 a	12.43 ± 0.05 b	12.89 ± 0.26 b	13.42 ± 0.08 a
(c)							
SE	His	1.29 ± 0.05 bc	1.34 ± 0.05 ab	1.40 ± 0.01 a	1.26 ± 0.01 b	1.31 ± 0.03 b	1.37 ± 0.01 a
	Arg	5.94 ± 0.17 ab	5.95 ± 0.25 ab	6.25 ± 0.06 a	5.94 ± 0.08 ab	5.82 ± 0.13 ab	6.08 ± 0.06 a
Total %		7.23 ± 0.21 ab	7.29 ± 0.30 ab	7.65 ± 0.06 a	7.20 ± 0.09 ab	7.13 ± 0.15 b	7.45 ± 0.06 a
(d)							
Total %		43.38 ± 1.61 bc	44.86 ± 1.64 ab	46.86 ± 0.19 a	42.67 ± 0.17 b	43.84 ± 0.9 b	45.74 ± 0.26 a


*NE, E, and SE stand for non-essential, essential and semi-essential amino acids, respectively. CM and IMS stand for conventional management practice and integrated management strategies (see Materials and Methods). The unit of proteinogenic amino acids is the percentage of dry mass. The means ± standard errors (*n* = 3) within each column followed by the same letter are significantly different (*P* < 0.05) according to Fisher’s least significant difference (LSD) test.*

**Table 4:** Results of ANOVA on the effects of year (Y), fertility levels (FL), integrated management strategies (M) and their interactions on protein content and proteinogenic amino acids in cotton embryos in 2012 and 2013.

Effect	*df*	Protein content	TAA	NE	E	SE	Glu	Lys	Met
Y	1	235.71**	0.64^ns^	1.07^ns^	0.55^ns^	0.01^ns^	0.93^ns^	0.64^ns^	2.35^ns^
FL	1	27.41**	0.11^ns^	0.07^ns^	0.46^ns^	0.01^ns^	0.08^ns^	0.14^ns^	2.54^ns^
M	2	45.82**	27.42**	30.48**	28.68**	12.75**	32.57**	24.59**	19.46**
Y × FL	1	2.03^ns^	6.35*	7.24*	4.38*	6.17*	7.17*	4.62*	5.71*
Y × M	2	0.68^ns^	0.48^ns^	0.27^ns^	0.31^ns^	2.46^ns^	0.32^ns^	0.29^ns^	0.06^ns^
FL × M	2	3.89*	5.00*	4.98*	4.42*	5.32*	4.95*	2.70^ns^	5.75*
Y × FL × M	2	0.70^ns^	5.27*	5.22*	4.73*	5.81**	5.20*	4.86*	3.18^ns^


*TAA, NE, E, and SE stand for total, non-essential, essential and semi-essential proteinogenic amino acids, respectively. *F* values and significance levels (^∗∗^*P* < 0.01, ^∗^*P* < 0.05 and ^ns^*P* ≥ 0.05). df, degree of freedom.*

**FIGURE 1:**
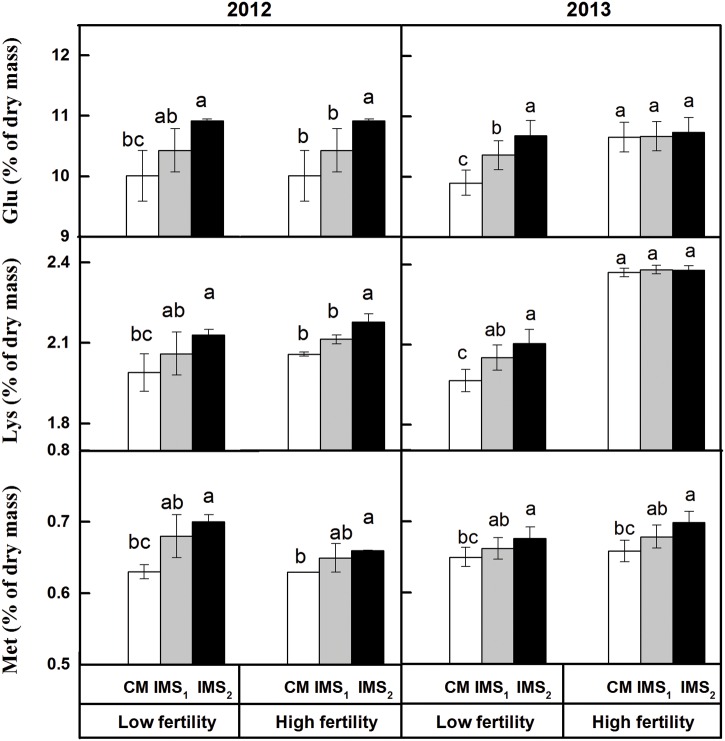
**Contents of Glu, Lys and Met in cotton embryos under conventional management practices and two integrated management strategies at two fertility levels in 2012 and 2013.** The data points represent the means, and the bars are the SEs. CM and IMS stand for conventional management practice and integrated management strategies (see Materials and Methods). Different letters indicate significant difference at the level of *p* < 0.05.

Glu, Lys, and Met are nutritional limiting factors for ruminants fed cottonseed kernel protein flour. The contents of Glu, Lys, and Met in IMS_2_ were increased by 4.7, 2.9, and 3.4% at a low fertility level and by 4.7, 1.5, and 4.0% at a high fertility level, respectively, compared to those embryos in CM (**Figure 1**). The ratio of each amino acid to the total proteinogenic amino acid content was relatively stable. These results indicate that the IMS increased the levels of proteinogenic amino acids but did not lead to an alteration in the level of any proteinogenic amino acid.

### Soil-Available Nitrogen Content

**FIGURE 2:**
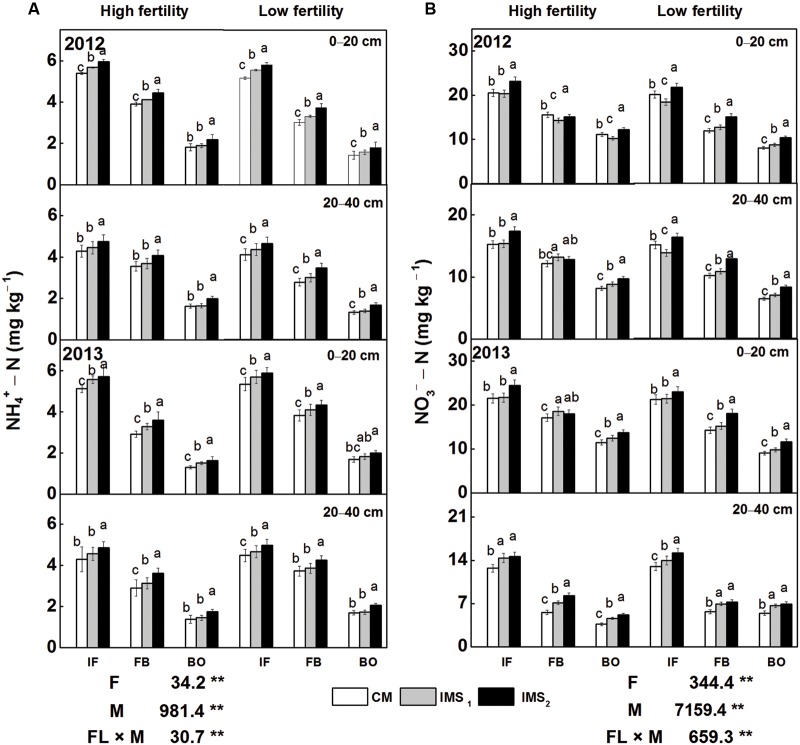
**Soil 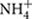 -N **(A)** and 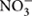 -N **(B)** levels under conventional management practices and two integrated management strategies at two fertility levels at soil depths of 0–20 cm and 20–40 cm in 2012 and 2013.** CM and IMS stand for conventional management practices and integrated management strategies (see Materials and Methods). IF, BF, and BO stand for the initial flowering stage, full-bloom stage, and boll-opening stage, respectively. Different letters indicate significant difference at the level of *P* < 0.05.

Regardless of the soil fertility levels or soil depth, the contents of 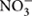 -N and 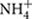 -N were consistently significantly higher in IMS_1_ and IMS_2_ than in CM at soil depths of 0–20 cm and 20–40 cm (**Figures 2A,B**). Furthermore, the 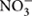 -N and 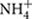 -N contents at the initial flowering stage were higher than those at the end of flowering and full-bloom stages. IMS_2_ exhibited the highest level of AV-N (sum of 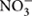 -N and 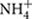 -N). Compared with CM, the contents of AV-N in IMS_1_ and IMS_2_ increased by averages of 6.37 and 16.86%, respectively, at soil depths of 0–20 cm and by 9.72 and 21.52%, respectively, at soil depths of 20–40 cm. These results indicated that soil N availability was significantly increased in both IMS_1_ and IMS_2_ compared with CM.

### GS and NADH-Dependent GOGAT in Developing Cotton Embryos

**FIGURE 3:**
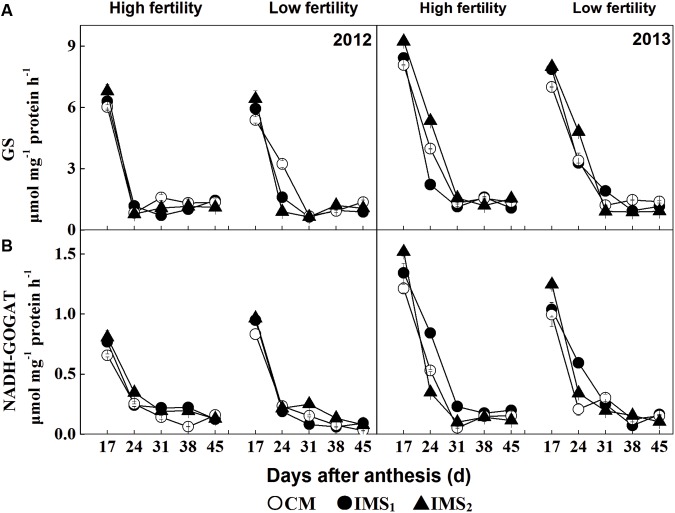
**Developmental profiles of glutamine synthetase **(A)** (GS, μmol γ-glutamyl-hydroxamate h^-1^ g^-1^ soluble protein) and **(B)** NADH-dependent glutamate synthase (GOGAT, μmol NADH h^-1^ g^-1^ soluble protein) for conventional management practices and two integrated management strategies at two fertility levels in 2012 and 2013.** For each sample point, we tested at least three independent samples. The data points represent the means, and the bars are the SEs. CM and IMS stand for conventional management practices and integrated management strategies (see Materials and Methods).

The enzymatic activities of GS and NADH-dependent GOGAT showed similar patterns of alterations, with their activities declining according to the duration of embryo growth in 2012 and 2013 (**Figure 3**). Compared with CM, GS activity increased during IMS_1_ and IMS_2_ embryo growth, with average increases of 4.5 and 13.6% at high fertility levels and of 11.2 and 16.5% at low fertility levels, respectively. Further, the NADH-dependent GOGAT activities in IMS_1_ and IMS_2_ increased by 13.9 and 24.1% at high fertility levels and by 9.1 and 20.6% at low fertility levels, respectively. These results indicated that the IMS enhanced the initial N assimilation in the developing cotton embryos.

### Free Amino Acid Supply and Protein Accumulation in Developing Cotton Embryos

**FIGURE 4:**
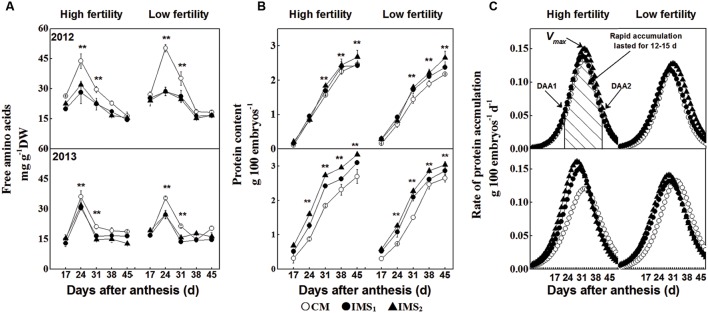
**The free amino acid contents **(A)** and protein accumulation **(B,C)** in cotton embryos under conventional management practices and two integrated management strategies at two fertility levels in 2012 and 2013.** The data points represent the means, and the bars are the SEs. Characterization of proteins accumulated in the cotton embryos was calculated using sigmoid growth curves (see Materials and Methods). CM and IMS stand for conventional management practice and integrated management strategies (see Materials and Methods). ^∗^ and ^∗∗^ denotes significant at the 0.05 level and 0.01 level, respectively.

Free amino acids are precursors to storage proteins and are incorporated into proteins during the growth of cotton embryos. The free amino acid content increased sharply after 17 DAA, rising from a very low level to a maximum level at 24 DAA and then declining (**Figure 4A**). This content decreased dramatically, accompanied by rapid protein accumulation in the cotton embryos after 24 DAA. Moreover, at 24 DAA, the free amino acid content was significantly higher in CM than in IMS_1_ or IMS_2_.

Sigmoid growth curves, used to quantify the rate of protein accumulation (g 100 embryos d^-1^), may reveal the reasons and key stages associated with the differences in protein content among integrated management treatments (**Figures 4B,C**). Rapid embryo protein accumulation began at 17–22 DAA and terminated at 32–37 DAA, lasting from 11 to 15 days in 2012 and 2013 (**Table 5**). The peak rate of protein accumulation was positively correlated with the protein content at maturity. Change tendency of the protein content was opposite to that of the free amino acids. Accumulation of a large amount of protein coincided with a rapid decrease in the free amino acid content, and evaluation of the different management treatments revealed that the protein content was limited by the protein accumulation rate rather than by the free amino acid content.

**Table 5:** Protein accumulation characteristics of cotton embryos under conventional management practices and two integrated management strategies at two fertility levels in 2012 and 2013.

Fertility levels	Integrated management strategies									Embryonic protein accumulation characteristics
		
		DAA_1_ (d)		DAA_2_ (d)		Duration (d)		Peak rate (g 100 embryos^-1^ d^-1^)		Protein content (g 100 embryos^-1^)	
						
		2012	2013	2012	2013	2012	2013	2012	2013	2012	2013
(a)											
High fertility	CM	21.6	20.5	33.5	35.4	11.9	15.0	0.134	0.120	2.35 b	2.97 c
	IMS_1_	21.0	18.6	32.9	32.0	11.9	13.4	0.139	0.150	2.43 b	3.07 b
	IMS_2_	21.7	17.2	33.7	30.8	12.0	13.5	0.150	0.161	2.68 a	3.30 a
	CV%	2.49	9.37	3.49	6.82	5.71	5.12	7.77	13.81	7.00	5.55
(b)											
Low fertility	CM	21.2	22.7	32.8	37.0	11.5	14.3	0.116	0.132	2.01 bc	2.63 b
	IMS_1_	19.8	19.1	33.2	34.0	13.4	14.9	0.118	0.132	2.38 b	2.91 ab
	IMS_2_	21.1	18.5	35.0	33.3	13.9	14.8	0.127	0.141	2.65 a	3.06 a
	CV%	7.47	9.56	4.88	4.94	7.92	1.85	4.11	8.54	13.81	7.65


*The characterization of protein accumulation in cotton embryos was calculated from **Figure 4B** (see Materials and Methods). CM and IMS represent the conventional management practices and integrated management strategies, respectively. DAA_1_ is the time at which embryonic protein accumulation began. DAA_2_ is the time at which embryonic protein accumulation ended. Duration is the difference in days between DAA_1_ and DAA_2_. CV% stands for coefficient of variation. The means within each column followed by the same letter are not significantly different (*P* < 0.05) according to the least significant difference (LSD) test.*

### Protein Quality and Relationships

The protein content was positively and significantly correlated with the content of soil available N, GS activity and peak rate of rapid protein accumulation, whereas it was negatively correlated with the free amino acid content (**Table 6**). Similar relationships were also observed for the levels of total proteinogenic amino acids, essential amino acids, semi-essential amino acids, Glu, Lys and Met.

**Table 6:** Correlations among protein traits, soil available N, N assimilation and characterization of protein accumulation.

Quality traits	Soil available N		GS activity	NADH-GOGAT activity	Free amino acid content	Duration	Peak rate
						
	0–20 cm	20–40 cm					
TAA	0.805**	0.708**	0.552*	0.173^ns^	-0.621*	-0.049^ns^	0.702**
E	0.772**	0.737**	0.471^ns^	0.092^ns^	-0.614*	-0.130^ns^	0.690**
SE	0.823**	0.736**	0.509*	0.131^ns^	-0.434 ^ns^	-0.152^ns^	0.601*
Glu	0.703**	0.639**	0.503*	0.156^ns^	-0.647**	-0.062^ns^	0.718**
Lys	0.572*	0.508*	0.516*	0.123^ns^	-0.618*	0.013^ns^	0.623*
Met	0.634**	0.528*	0.493^ns^	0.125^ns^	-0.575*	-0.108^ns^	0.752**


*TAA, E, and SE stand for total, essential and semi-essential proteinogenic amino acids, respectively. Glu, Lys, and Met stand for glutamic acid, lysine, and methionine, respectively. Soil available N represents the sum of the 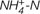 and 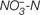 levels. The duration and peak rate of protein accumulation were calculated from sigmoid growth curves (see Materials and Methods). ^∗^ and ^∗∗^ denote significant at the 0.05 level and 0.01 level, respectively.*

**FIGURE 5:**
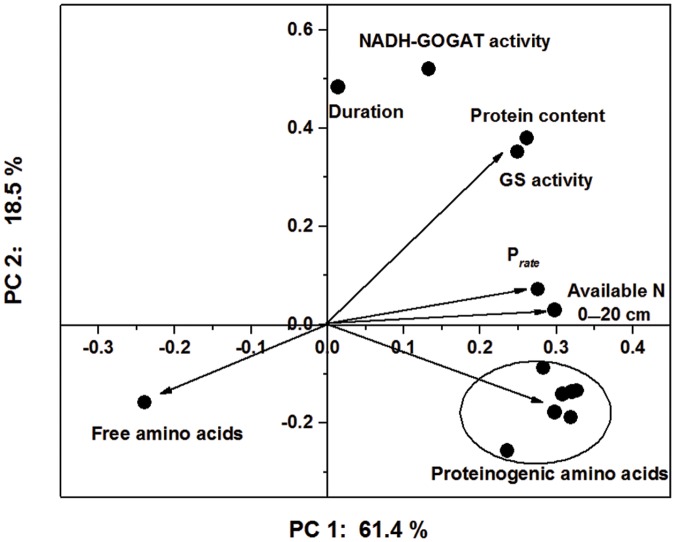
**Principal component analysis of the soil-available N content, N assimilation, rate of protein accumulation and proteinogenic amino acid profile based on the first two principal components (PC 1: principal component 1; and PC 2: principal component 2).** The arrows represent the principal component loadings, and the ellipses represent the proteinogenic amino acid profiles.

**FIGURE 6:**
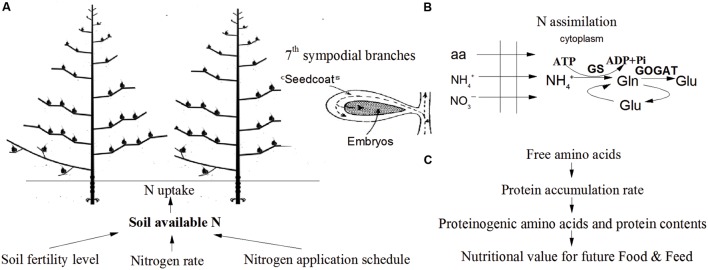
**Schematic overview of the integrated management strategies on protein quality of cotton embryos. (A)** Integrated management strategies increases the soil available N content. **(B)** Nitrogen uptake, translocation and assimilation were enhanced by soil available N content. **(C)** Cotton embryonic protein content was limited by the conversion of free amino acids into storage protein rather than by the free amino acid content.

In PCA, the first two principal components explained 61.4% and 18.5% of the total variance in the sample set, respectively, whereas the third principal component only accounted for 7.5% of the variance (**Figure 5**). Essential amino acids, semi-essential amino acids, Glu, Lys and Met were clustered as a group in the score plot. The content of soil available N and the peak rate of protein accumulation were most closely related to the proteinogenic amino acid profiles in the score plot. These results indicated that the improvements in the nutritional value of the cotton embryos observed with use of the IMS could be attributed to increases in soil N availability, the initial step of N assimilation and the conversion of free amino acids into storage proteins (**Figure 6**).

## Discussion

The initial question that motivated us to perform this study was whether matching a high-yielding variety with IMS would prove useful in improving the nutritional value of cotton embryos. We were also particularly interested in the following questions: What improves their nutritional value? What is the physiological basis of high quality? What are the guidelines for increasing crop quality in other cotton-producing regions?

Most of the previous studies are focusing on the developing high fiber yield and optimal fiber propriety through crop breeding (Cai et al., 2014; Guan et al., 2014; Ning et al., 2014), stress-resistance cultivation (Kawakami et al., 2012; Liu et al., 2015; Kuai et al., 2016), and crop management (Kawakami et al., 2012). However, high-quality and nutritional value-added cottonseed by improving management practices can increase crop yields and improve farmers’ livelihoods dramatically. In this study, the levels of proteins, essential amino acids, and semi-essential amino acids, especially those of Glu, Lys, and Met, were higher in IMS_1_ and IMS_2_ embryos than in CM embryos (**Tables 2** and **3**; **Figure 1**). In addition, fiber yield, seed yield, seed weight were also increased dramatically in IMS_1_ and IMS_2_ treatments (data not given). The impact of IMS on cotton embryo protein quality was more obvious at a low soil fertility level than at a high soil fertility level (**Tables 2** and **3**). Previously it has been reported that ruminants fed with cottonseed, like cows produce more milk and its protein content were also increased (Bertrand et al., 1998; Meyer et al., 2001; Taghizadeh et al., 2005). Therefore, an increase in the proteinogenic amino acid content achieved by adopting IMS resulted in enhanced nutritional value of the cotton embryos.

Nitrogen fertilization rate, plant density, and plant growth regulators have been shown to have a positive effect on cottonseed oil and protein contents (Sawan et al., 1993, 2006). In this study (using IMS), the N rate and fertility level contributed to the increase of protein content (**Table 2**), are in agreement with the results of the previous single-factor experiments (Chen et al., 2015), suggesting that protein content were increased with increasing N fertilization rate (Sawan et al., 1988; Egelkraut et al., 2004). As the plant density increased, the protein content were also increased (Rafiq et al., 2010), and it would not need an additional labor input because of mechanized transplanting. The N rate in IMS_1_ was an economic fertilization rate as reported previously (Chen et al., 2016) and it was higher compared with those reported at the other experimental stations (Rochester et al., 2001) might be attributed to the saline-alkali soil (pH 8.26–8.47). Salt stress limits plant growth (Zhang et al., 2015) and therefore the high economic fertilization N rate (375 kg ha^-1^). Further, increase in the application of N at flowering and boll-forming stage not only increased cotton yield and biomass (Yang et al., 2011), but also proved to be useful for increasing the protein content in cotton embryos (**Table 2**). Sufficient N supply from flowering to boll forming stage was the main reason for the increment of protein content. Additionally, this study also found that the substrate seedling-raising method contributed to increase in protein content. The probable reason behind this was that bio-organic fertilizer bring the efficacious living-cell into a soil and then affect the N availability in the soil (Inselsbacher et al., 2010). Due to unusual weather changes in recent years, temperature and photosynthetically active radiation in 2013 was higher than 2012, like cumulative photo-thermal product increased by 30.8% during the seed growth. Seed weight was increased dramatically; therefore, the protein content (g 100 embryos^-1^) was higher than the year in 2012. Therefore, a set of the best management practices for cotton production was integrated that could increase protein quality in cotton embryos for feed purposes.

A study using a method based on isotope (^15^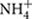 and ^15^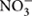) labeling has demonstrated that soil N is ultimately translocated into seeds (Kullmann and Geisler, 1986), suggesting a potential contribution of soil N to the protein content of seeds. Embryos take up N, carbohydrates and free amino acids from seed coats (De Ruiter et al., 1986) and use them for embryo growth and the synthesis of storage proteins. Based on these studies, it appears that the soil N and seed protein contents are closely interrelated. In this study, as shown in **Figure 2** and **Table 2**, the soil-available N and protein contents were higher in both IMS_1_ and IMS_2_ compared with that in CM. Similar results have been reported in tomato (Javaria and Khan, 2011) and potato (Zotarelli et al., 2015), suggesting that rate and timing of nitrogen fertilizer application increases the soil-available N content. Furthermore, principal component and Pearson’s correlation analyses revealed that the soil-available nitrogen content was positively correlated with the nutritional value of cotton embryos and that the soil-available nitrogen and proteinogenic amino acid profiles were closely matched in a score plot (**Table 6**; **Figure 5**). Therefore, high soil-available nitrogen content contributes to the nutritional value of cotton embryos.

A study using the isotope (^15^N) labeling also has shown that GS and NADH-GOGAT are correlated with N assimilation and re-distribution (Zhang et al., 2014). In this study, comparisons of management treatments at two fertility levels demonstrated that the protein content (g 100 embryos^-1^) was positively correlated with the GS and NADH-dependent GOGAT activities (**Table 6**). Both soil N and GS activity increased protein content in cotton embryos (**Figures 2** and **3**; **Table 2**), that is consistent with previous research findings, suggesting that 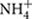-N and 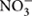-N concentrations in the soil tightly regulate the GS and GOGAT activities (Zhao and Shi, 2006), and that GS activity and actual accumulation of reduced N are closely interrelated in cotton (Radin et al., 1975) and wheat (Brunetti and Hageman, 1976). Therefore, a high level of soil available N increases N assimilation in developing cotton embryos (**Figures 6A,B**). Furthermore, GS plays a key role during the early stage of embryo growth.

A previous study reported that the synthesis of embryonic proteins is regulated by the supply of free amino acids (Takahashi et al., 2003; Hernandez-Sebastia et al., 2005). In this study, the free amino acid content decreased simultaneously with accumulating storage protein after 24 DAA (**Figures 4A,B**). The protein accumulation rate was higher in IMS_1_ and IMS_2_ embryos over CM, meanwhile the amino acids content showed the opposite trends, indicating that the protein content was limited by the protein accumulation rate rather than by the free amino acid content. Furthermore, the peak protein accumulation rate was positively associated with the protein content at maturity, exhibiting a period of rapid protein accumulation during 24–38 DAA (**Figure 4C**, **Table 6**), whereas the free amino acid content peaked at 24 DAA (**Figure 4A**). It seemed that embryos show an early period for free amino acids accumulation, which was characterized by large amount of free amino acid accumulation during 17–24DAA (**Figure 4A**). The embryos accumulate almost 75% of the total protein within rapid protein accumulation stage for a short period of time (lasted for 12–15 days) that was attributed to an early period of free amino acids accumulation. Similar study also observed in other researches, for instance in soybean (Rotundo et al., 2011), suggesting protein content was determined by different combinations of rate and duration of content accumulation (**Figure 6C**).

The indeterminate growth habit of cotton results in the formation of bolls over a range of time as the plant grows and under different environmental and nutritional conditions (Bondada and Oosterhuis, 2001). In this study, the soil available N decreased with plant growth, so, late season boll are susceptible to low soil N. This could explain why protein content in upper fruiting branches was higher than middle and lower fruiting branches (Chen et al., 2015). Therefore, adding extra N and allocating N to flowering and boll forming stage are beneficial to soil available N for the formation of late season bolls and it might increase the uniformity of cottonseed. Previous results also suggest adding extra potash fertilizer or allocating it for the development of late season bolls might result in longer fibers in upper fruiting branches (Yang et al., 2016). To sum up, whether adding extra nitrogen and potash fertilizer, and allocating these fertilizer to the flowering and boll formatting stage could result in both longer fiber and high seed protein quality still further results.

## Conclusion

A set of best management practices for cotton embryonic protein, collectively known as IMS, was assembled. The combined application of the economic N rate, growth-driven N application schedule, a high plant density, and seedling raising with bio-organic matter markedly improved protein quality, especially the levels of Glu, Lys, and Met. Increased protein quality was attributed to a high level of soil-available N content. Soil 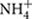 -N and 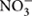-N transported into seed and then utilized through GS and GOGAT pathway that are beneficial for N assimilation in developing cotton embryos. Reduced N and incorporated into carbon skeletons of storage protein was determined by the rate and duration of protein accumulation. The key factor limiting the protein content was the protein accumulation rate rather than the free amino acid content. Therefore, selection of a high-yielding cultivar (SIZA-3) adapted to the cotton production region, transplantation of seedling with bio-organic fertilizer, a high plant density (30000 plant ha^-1^), and an economic N fertilization rate (375 kg ha^-1^, 20% applied as a basal fertilizer, 25% at the initial flowering stage, 40% at the full-bloom stage and 15% at the end of the flowering stage) are highly recommended for cotton management to promote protein quality of cotton embryos for ruminants feed (**Figure 6**).

## Author Contributions

ZZ, YM, and BC conceived the idea and led the study design. HY carried out the experiment, performed analysis and wrote the paper. XZ assisted in plant sampling and laboratory analysis. WZ and YW assisted manuscript writing and editing. All authors reviewed the manuscript.

## Conflict of Interest Statement

The authors declare that the research was conducted in the absence of any commercial or financial relationships that could be construed as a potential conflict of interest.

## Abbreviations

AV-N: soil available nitrogen
CM: conventional management practices
Glu: glutamate
GS: glutamine synthetase
IMS: integrated management strategies
Lys: lysine
Met: methionine
NADH-GOGAT: NADH-dependent glutamate synthase
